# Characterization of a restriction modification system from the commensal *Escherichia coli *strain A0 34/86 (O83:K24:H31)

**DOI:** 10.1186/1471-2180-8-106

**Published:** 2008-06-27

**Authors:** Marie Weiserová, Junichi Ryu

**Affiliations:** 1 Institute of Microbiology, v.v.i., Academy of Sciences of the Czech Republic, Vídeňská 1083, 142 20 Prague 4, Czech Republic; 2 Department of Biochemistry and Microbiology, Loma Linda University, Loma Linda, CA92350, USA

## Abstract

**Background:**

Type I restriction-modification (R-M) systems are the most complex restriction enzymes discovered to date. Recent years have witnessed a renaissance of interest in R-M enzymes Type I. The massive ongoing sequencing programmes leading to discovery of, so far, more than 1 000 putative enzymes in a broad range of microorganisms including pathogenic bacteria, revealed that these enzymes are widely represented in nature. The aim of this study was characterisation of a putative R-M system EcoA0ORF42P identified in the commensal *Escherichia coli *A0 34/86 (O83: K24: H31) strain, which is efficiently used at Czech paediatric clinics for prophylaxis and treatment of nosocomial infections and diarrhoea of preterm and newborn infants.

**Results:**

We have characterised a restriction-modification system EcoA0ORF42P of the commensal *Escherichia coli *strain A0 34/86 (O83: K24: H31). This system, designated as EcoAO83I, is a new functional member of the Type IB family, whose specificity differs from those of known Type IB enzymes, as was demonstrated by an immunological cross-reactivity and a complementation assay. Using the plasmid transformation method and the RM search computer program, we identified the DNA recognition sequence of the EcoAO83I as GGA(8N)ATGC. In consistence with the amino acids alignment data, the 3' TRD component of the recognition sequence is identical to the sequence recognized by the EcoEI enzyme. The A-T (modified adenine) distance is identical to that in the EcoAI and EcoEI recognition sites, which also indicates that this system is a Type IB member. Interestingly, the recognition sequence we determined here is identical to the previously reported prototype sequence for Eco377I and its isoschizomers.

**Conclusion:**

Putative restriction-modification system EcoA0ORF42P in the commensal *Escherichia coli *strain A0 34/86 (O83: K24: H31) was found to be a member of the Type IB family and was designated as EcoAO83I. Combination of the classical biochemical and bacterial genetics approaches with comparative genomics might contribute effectively to further classification of many other putative Type-I enzymes, especially in clinical samples.

## Background

Classical restriction and modification (R-M) systems provide the host bacteria with protection against infection by foreign DNA and protect the cellular DNA from restriction by methylation of adenosyl or cytosyl residues within the sequence recognised by the restriction enzymes. These enzymes are divided into three groups; Type I, II and III, of which the Type I R-M systems are the most complex discovered so far.

Genomic sequencing still detects new putative R-M systems, and comparative genomics reveals that R-M systems are spread widely in bacteria and archea [1]. R-M systems are also encoded by algal viruses as well as bacteriophages [2]. Almost 3800 R-M systems have been characterized to date and more than 4000 have been predicted from bioinformatic analyses of DNA sequences [1]. Based on functional complementation, antibody cross-reactivity, sequence similarity and specific distance between methylated adenines, Type I R-M systems are divided into four families (Type IA e.g. EcoKI, Type IB e.g. EcoAI, Type IC e.g. EcoR124I, ID e.g. StySBLI) [3-5]. KpnBI R-M system was detected as the prototype of a new family IE [6]. In these most complex R-M systems, restriction and modification activities are catalysed by one enzyme composed of three different subunits, which are encoded by the *hsdR, hsdM *and *hsdS *genes. Except for plasmid-borne R-M systems, most of the *hsd *genes are chromosomally located alleles, especially in enteric bacteria in a locus at 98.6 min termed the immigration control region (ICR). ICR was first defined in *Escherichia coli *K-12 and includes, within 14 kb of DNA, the *hsdR*, *hsdM *and *hsdS *genes encoding the EcoKI Type I system, as well as the methylation-dependent restriction system genes, *mcrB*, *mcrC *and *mrr *[7]. Type I restriction genes resident here (linked to *serB *and *thr*) are known to be highly variable in specificity, both within *E. coli *and among enteric bacteria [3].

Comparative bacterial genomics of the ICR sequence, including its boundary, revealed the same framework genes in the same order, interrupted by a variable region in the same location in other strains of enteric bacteria. In addition to the well-studied *E. coli *laboratory strain K-12, these strains included another *E. coli *laboratory strain (W), the *E. coli *pathogens CFT073, O157:H7 and K1, and *S. typhimurium *LT2. In all the strains, the ICR is flanked by the genes *yjiS *and *yjiA *[8]. The authors provided evidence that the ICR plays the role of a replaceable cassette contributing to variation of restriction enzyme genes in *E. coli *and its relatives. More recently, the ICR was found in *E. coli *strain A0 34/86 (O83: K24: H31). This strain is efficiently used in the prophylaxis and treatment of nosocomial infections and diarrhoea of preterm and newborn infants in Czech paediatric clinics [9,10]. Analysis of its genome, by bacterial artificial chromosome (BAC) library cloning, revealed that among 100 examined BAC clones covering the A0 34/86 genome, one (BAC C4/1) reproducibly conferred on the laboratory strain DH10B an enhanced capacity to persist in the intestine of newborn piglets. Sequencing revealed that this BAC clone carried genes encoding, among others, a putative restriction-modification system Type I [11], identified in **REBASE **[1] by the ORF as EcoA0ORF42P and designated EcoAO83I.

In this paper, we characterized this restriction system by combination of both classical genetics and comparative genomics. We demonstrated the family affiliations based on the strictest requirement for membership of a family; the complementation test and antibody-cross reactivity. Using a strategy employing a unique collection of pL and pE plasmids transformation method along with the RM search computer program [12], the DNA recognition sequence of the EcoAO83I R-M enzyme was determined.

## Results and Discussion

### Comparative genomics

The *hsd *genes of the predicted Type I R-M system EcoA0ORF42P were found on a fragment of approximately 10 kb of the C4/1 BAC; a location similar to the *hsd *genes of the enterohaemorrhagic (EHEC) O157: H7 and uropathogenic (UPEC) CFT073 strains. This region corresponds to the ICR and it is obvious that these *hsd *genes are allelic [11].

The sequence data and BLASTP results available on **REBASE **[1] permit a preliminary characterization of the R-M system from the *Escherichia coli *strain A0 34/86 (O83: K24: H31) by comparison of the amino acid identity with the R-M systems from (EHEC) O157: H7 [13] and (UPEC) CFT073 [14]. The appearance of Type IB enzymes EcoAI and EcoEI [5] on the list of "closest neighbours" strongly suggested that the analysed systems could belong to the same family. The level of identity of both HsdR and HsdM subunits is more than 90%, which is in agreement with the defined rules for the family membership [15]. The lower level of identity with the HsdR of EcoEI is the known exception [4]. Comparison with the EcoKI (Type IA) is given for illustration of the low interfamily levels of amino acid identity of HsdR (17 to 26%) [16] and HsdM (from 25 to 33%) subunits [15] (Table 1).

**Table 1:** Sequence comparisons of HsdR and HsdM subunits

	**EcoA0ORF42P**	
	
**R-M system**	HsdR	HsdM
**CfrAI**	NA	72/62 aa^1^
**EcoAI**	99	98
**EcoEI**	77	90
**StySKI**	92/219 aa	96
**EcoO157ORF5947P**	99	99
**EcoKO157ORF5307P**	99	99
**EcoCFTORF5424P**	99	99
**EcoKI**	26	28

The R-M systems CfrAI, EcoAI and EcoEI are prototypes of Type IB family, StySKI identifies the first member of the IB family in *Salmonella *species [38], EcoKI is a prototype member of Type IA [39]. The values are the per cent identity of aligned sequences. EcoA0ORF42P is from *E. coli *A0 34/86 (O83: K24: H31), EcoO157ORF5947P is from *E. coli *O157:H7, EcoKO157ORF5307P is from *E. coli *O157:H7 Sakai strain [40] and EcoCFTORF5424P is from *E. coli *CFTO73. P means putative R-M system, NA – the sequence is not available, ^1 ^alignment of the number of aa so far available

Comparison of the HsdS subunits (Table 2) revealed strong similarities in the conserved regions, while sequences of TRD regions, responsible for recognition of the specific sites on DNA, differ significantly. The higher identity between TRD2 of S.EcoEI and S.EcoA0ORF42P indicates that the 4 bp component of their bipartite recognition site might be either very similar or identical. This amino acid alignment also revealed the direct repeat typical of Type IB HsdS subunits [17,18]. The first repeat starts 50 aa and the second 325 aa from the N-terminus.

**Table 2:** Sequence comparison based on S.EcoA0ORF42P

**HsdS subunit**	**Per cent identity in regions**^a^					**Recognition site**^b^
		
	**N-cons**	**TRD1**	**Central cons**	**TRD2**	**C-cons**	
**S.EcoEI**	80	26	86	72	83	GAGN(7)ATGC
**S.EcoO157ORF5947P**	**91**	**30**	**89**	**21**	**84**	
**S.EcoKO157ORF5307P**	**91**	**30**	**89**	**21**	**84**	
**S.EcoAI**	**88**	**26**	**86**	**16**	**76**	GAGN(7)GTCA
**S.EcoCFTORF5424P**	**88**	**26**	**85**	**16**	**76**	
**S.CfrAI**	85	27	85	24	84	GCAN(8)GTGG
**S.StySKI**	83	29	85	23	76	CGATN(7)GTTA

^a ^The numbers reflect the per cent identity in each of the regions recalculated on the basis of the alignments given by BLAST in **REBASE **[1] for the entire sequence of S.EcoA0ORF42P. The conserved (cons) and variable (TRD = target recognition domain) regions for Type IB are defined according to Kannan et al. [17]. ^b ^Only known recognition sequences are shown.

Based on the sequence alignment, we conclude that the R-M system predicted in *E. coli *A0 34/86 (O83: K24: H31) is the new member of Type IB family, but is not an isoschizomer of either any prototype of the Type IB members or any sequenced putative IB R-M systems. Moreover, this comparison revealed that the putative R-M systems EcoO157ORF5947P and EcoKO157ORF5307P are identical and, together with EcoCFTORF5424P, also should belong in the IB family.

### Analysis of restriction and modification activities

First, we analysed whether the putative R-M system EcoA0ORF42P is functional. Individual *E. coli *DH10B clones harbouring BAC C4/1, plasmid pFFP30 (EcoAI) and pGC1 (EcoEI) were tested for restriction activity using λ.*vir *(Table 3).

**Table 3:** Analysis of the specificity of the *E. coli *A0 34/86 (O83: K24: H31) restriction system

**Plasmid (R-M system)\Phage λ.specificity**	λ.0	λ.A	λ.E	λ.C4/1
BAC (none)	1	1	1	1
pFFP30 (EcoAI)	2 × 10^-3^	1	3 × 10^-2^	1 × 10^-2^
pGC1 (EcoEI)	1 × 10^-3^	4 × 10^-2^	1	6 × 10^-2^
C4/1 (EcoA0ORF42P)	2 × 10^-4^	1 × 10^-4^	5 × 10^-4^	1

Individual *E. coli *DH10B clones harbouring BAC C4/1 with EcoA0ORF42P, plasmid pFFP30(EcoAI) and pGC1(EcoEI) were tested for restriction of λ.*vir *0 (phage with no modified sites on its DNA), phage λ.*vir *specificity of EcoAI, EcoEI and C4/1 were produced on DH10B [pCFF30], DH10B [pGC1] and DH10B [BAC C4/1], respectively. The numbers represent the efficiency of plating (e.o.p.) of lambda phage

The plating efficiency of the phage was high only on the strain that carried the HsdS subunit of the same specificity as its previous host. This result shows that the system restricts not only λ.0, but also λ.A and λ.E. On the other hand, the phage modified on specificity EcoA0ORF42P (C4/1) was restricted by strains with R-M systems of specificity EcoAI and EcoEI.

Thus, the BAC C4/1 encodes a functional, Type IB related restriction system, whose target sequence is present on lambda DNA but differs from those of known IB enzymes. Consequently this R-M system was named EcoAO83I.

### Complementation analysis

The most important rule for estimation of a membership of the same Type I family is the complementation test. Type I R-M systems detected in *Lactococcus *could be separated into two families according the sequence analysis, showing only 42.2% and 37.3% identity of HsdM and HsdR subunits, respectively. Nevertheless, as assessed by complementation analysis [19], they belong to one family.

The complementation test is based on the fact that the HsdR and HsdM subunits are interchangeable within the members of a family. We used this test successfully for complementation between EcoKI and EcoBI enzymes in analysis of temperature sensitive mutants of the EcoKI HsdS subunit. These tests were performed *in vivo *using a partial diploid, in which one set of *hsd *genes was on the chromosome and the second set was provided by an F' plasmid, or *vice versa *[20].

In this study, the partial diploids were prepared by transforming the BL21(DE3) strains with a BAC C4/1 carrying the *hsdR, hsdM *and *hsdS *genes of EcoAO83I and with plasmids carrying the *hsdS *and *hsdM *genes of EcoAI. In complementation experiments, where the HsdS subunits of different specificities are produced in the presence of HsdR and HsdM, there should be a competition between these two HsdS subunits for assembly into an endonuclease. The strain should express restriction and modification functions of both of the two specificities. As expected, the HsdR and HsdM subunits of EcoAO83I substituted for the HsdR and HsdM subunits of EcoAI, as evident from the presence of the two specificities detected after *E. coli *transformation with plasmids BAC C4/1 and pJP21 or pJP24 (see Additional file 1). It should be pointed out that competition of HsdS EcoAI (on plasmid pJP24) for missing subunits is more successful when the HsdM subunit is also present (on plasmid pJP21). Competition of MTase for the HsdR subunit only results in a more efficient restriction of phage λC4/1. Conversely, assembly of sole HsdS EcoAI subunit with HsdM EcoAO83I obviously causes an imbalance of the subunits for assembly of EcoAO83I REase, resulting in a two orders of magnitude lower efficiency of restriction of phages λ.0 and λ. A. This complementation test confirmed the allocation of EcoAO83I to the Type IB family.

### Antibody cross reactivity

Antibodies raised against a representative of a known family of R-M enzymes can be very effectively used for serological screens of cell extracts with putative restriction enzymes. Antibody cross reactivity is also one of the most strict requirements for membership of a family [21].

**Figure 1:**
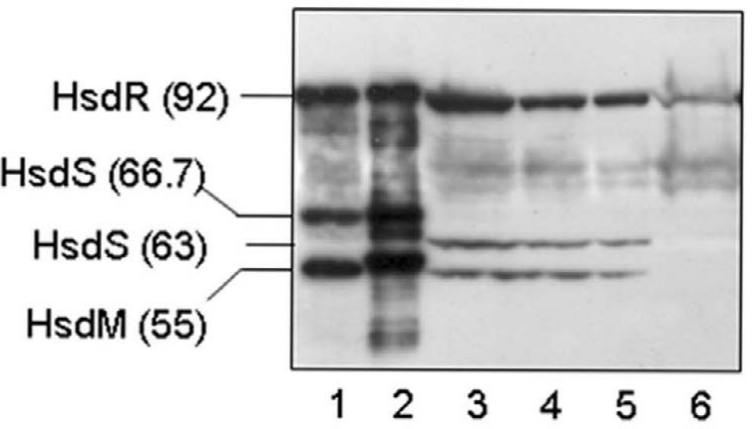
**Detection of Hsd polypeptides in total cell extracts using the anti-EcoAI antibody**. lane 1, purified EcoAI enzyme – standard; lane 2, extract from *E. coli *DH10B [pFFP30] expressing the EcoAI system; lanes 3, 4, extracts from *E. coli *DH10B [BAC-C4/1] expressing the EcoAO83I; lane 5,*E. coli *A0 34/86 (O83: K24: H31), EcoAO83I expressed from the chromosome; lane 6,*E. coli *DH10B [BAC] – control with no R-M system; The position of the Hsd subunits is indicated, molecular weights in kD are in bracket.

Proteins of cell-free extract prepared from the bacterial clone DH10B [BAC C4/1] harbouring the plasmids with *hsd *genes coding for EcoAO83I were separated by SDS-PAGE and transferred to a nitrocellulose membrane followed by immunoassay analysis using rabbit polyclonal antibodies against EcoKI, EcoAI, and EcoR124I – representatives of IA, IB, and IC families, respectively. No immunological cross-reactivity was observed in the experiments with anti-EcoKI and anti-EcoR124I antibodies (data not shown), while Hsd subunits were clearly detected by anti-EcoAI antibody. The EcoAO83I subunits were expressed from chromosomally located *hsd *genes in the original *E. coli *AO43/86 083 strain as well as from genes cloned onto BAC C4/1 (Fig. 1). Immunodetection also revealed that the HsdS subunit of EcoAO83I is smaller than the HsdS of EcoAI.

### Identification of the specific recognition sequence

To identify the recognition sequence of the EcoAO83I enzyme, a total of 38 plasmids were used for transformation (see Additional file 2). The relative efficiency of transformation (EOT) for DH10B [BAC-C4/1] versus DH10B was calculated. Plasmids exhibiting EOT values lower than 0.1 were assumed to contain one or more recognition sites [22]. Analysis of these data with the RM search program [12] revealed only one possible candidate sequence, GGA(8N)ATGC, without any degeneracy. The 3' TRD component of the recognition sequence is identical to EcoEI, which is consistent with the aa alignment data (Table 2).

**Figure 2:**
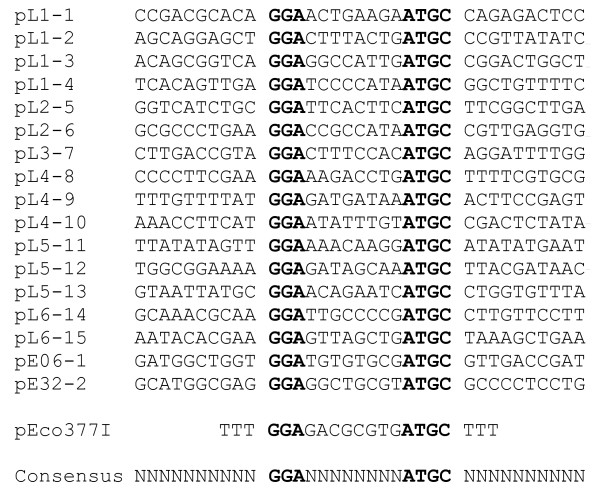
**Specific recognition site of the EcoAO83I R-M enzyme**. EcoAO83I site, GGA(8N)ATGC and surrounding DNA sequences on the pL series lambda clones and two pE series clones. pEco377I [22] contains 20 mer oligoduplex shown above, which is cloned at the unique EcoRV site of pMECA [24]

This sequence exists 15 times in phage lambda DNA and is shown with the surrounding bases in Fig. 2. The sequences show that the 8N portion of the recognition sequence is completely random. The abundance of the target sites explains the strong restriction of phage lambda (Table 3 and Additional file 1) [23].

The recognition sequence revealed here is identical to the previously reported prototype sequence for Eco377I [22]. To confirm the predicted recognition sequence, plasmid pEco377I was used for transformation. The plasmid contains the predicted sequence in a 20 mer oligoduplex (Fig. 2) cloned at the EcoRV site of pMECA [24]. As shown in Additional file 2, pEco377I was restricted to the 10^-3 ^level. To examine the modification status of the plasmids, two plasmids were recovered from the transformation plates. The transformants showed complete modification (EOT = 1.0 and 1.1) on DH10B [BAC-C4/1] cells. The plasmid R-M tests confirmed that the EcoAO83I enzyme recognizes and modifies the same target sequence as the Eco377I, which strongly supports the pertinence of Eco377I prototype to the Type IB family. Since there is only one adenine on each side of the recognition sequence available for methylation, we assumed that these adenines are the targets for methylation. In this case, the distance between adenines is 9, and corresponds to the Type IB family definition [4,25].

Recently, the sequence of another isoschisomer of EcoAO83I appeared in **REBASE **[1]. This putative R-M system, Eco536ORF4677P from *Escherichia coli *536 [26], shares 99% aa identity not only between HsdM and HsdR subunits but also between HsdS subunits. Thus, it is very likely that this putative R-M system is also an isoschizomer of prototype Eco377I. It remains to elucidate how broadly this system is spread among *E. coli *strains.

## Conclusion

Putative R-M system EcoA0ORF42P in the commensal *E. coli *strain A0 34/86 (O83: K24: H31) is a functional member of the Type IB family and was designated as EcoAO83I. DNA recognition sequence of EcoAO83I was identified as GGA(8N)ATGC, identical to the previously reported prototype sequence for Eco377I and its homologues, which in reverse, allowed us to classify these systems as new members of the Type IB family. The 3' TRD component of the recognition sequence is identical to EcoEI, which is consistent with the aa alignment data.

GGANNNNNNNN**ATGC **(EcoAO83I)

 GAGNNNNNNN**ATGC **(EcoEI)

Contribution of the described R-M system to the enhanced persistence of the appropriate clone in the porcine intestine as a model is to be analysed. Combination of the classical biochemical and bacterial genetics approaches with comparative genomics might contribute effectively to further classification of many other putative Type I enzymes.

## Methods

### Bacterial strains, plasmids and microbiological techniques

Table 4 lists the *Escherichia coli *strains and plasmids used in this study. Cells were grown in Lysogeny Broth (LB) [27] medium with addition of antibiotics (ampicillin; 100 μg ml^-1^, chloramphenicol; 50 μg ml^-1^) where required. Transformation and manipulation of nucleic acids were performed as described in [28]. BAC C4/1 was introduced into the appropriate strains by electroporation (Biorad Gene Pulser: 2.5 kV, 25 μF, 200 Ω). The virulent mutant of phage λ (λ.*vir*) was used for testing the restriction and modification.

**Table 4:** Bacterial strains and plasmids

***E. coli* strains**	**Relevant characteristics**	**Reference**
C122	prototroph, Δ*hsd*	British Culture Collection Strain No. 122
BL21(DE3)	F^- ^*dcm*, *ompT hsdS *(r_B_- m_B_-) *gal λ *(DE3)	[34]
DH10B	F^- ^– *mcrA Δ *(*mrr-hsdRMS-mcrBC*) phi 80*lacZ*DM15 *Δ lacX74 recA1 endA1 ara D139 Δ(ara, leu)7697 galU galK rpsL nupG*	Invitrogen
A0 34/86	serotype O83: K24: H31	[9,10]
DB24	*dcm-6 dam-16::Kan mcr-62 Δ(mcrB-hsd-mrr)114 *	[33]

**Plasmids**		

BAC	pBeloBAC11 cloning vector: ori2 (IncFI), designed for the construction of Bacterial Artificial Chromosomes (BACs), Cm ^R^	[35]
BAC C4/1	about 56 kb insert from strain *E. coli *A0 34/86 (O83: K24: H31) into pBeloBAC11	[11]
pL series	series of pMECA plasmids containing lambda DNA fragments, Ap^R^	[22]
pE series	*E. coli *K-12 chromosomal fragments subcloned into the pUC9 vector, Ap^R^	[32]
pEco377I	pMECA plasmid with pEco377I recognition sequence at EcoRV site	[22]
pUC19	cloning vector, Ap^R^	[36]
pGC1	*hsdR, hsdM *and *hsdS *genes of the EcoEI R-M system, Ap^R^	[5]
pFFP30	*hsd *region of EcoAI on HindIII fragment cloned into pBR322, Ap^R^	[5]
pJP24	HincII/NotI fragment from pJP21 carrying the *hsdS *of the EcoAI R-M system cloned into NdeI/NotI pET32a under control of the P_*T7g10 *_promoter. Ap^R^	[37]
pJP21	A derivative of pFFP30 and pET32a carrying the *hsdM *and *hsdS *genes of the EcoAI R-M system under control of the P_*T7g10 *_promoter. Ap^R^	[37]

Partial diploids for the complementation test were prepared by electroporation of BAC C4/1 carrying the *hsdR, hsdM *and *hsdS *genes of EcoA0ORF42P into the BL21(DE3) strains, followed by transformation with plasmids pJP21 and pJP24 carrying the *hsdS *gene or *hsdS *and *hsdM *of the EcoAI R-M system, respectively. The standard plating assays described previously [29] were used for restriction and modification tests.

### Preparation of total cell extract and immunodetection

To prepare total cell proteins, aliquots of bacteria were harvested, resuspended in SDS sample buffer and boiled for 5 min. Equal amounts of solubilized proteins were separated by SDS-PAGE [30] and transferred to a nitrocellulose membrane in CAPS buffer, pH 11, using a semi-dry blotter (Sigma). Hsd polypeptides were identified by rabbit polyclonal antibodies anti-EcoKI, anti-EcoAI and anti-EcoR124I [31] according to the standard Western blotting protocol using Super Signal West PicoChemiluminescent Substrate (Pierce).

### Determination of the recognition sequence

The recognition sequence of the EcoAO83I enzyme was determined using the CaCl_2_-heat shock plasmid transformation method [22] and the RM search computer program [12]. DH10B and DH10B [BAC-C4/1] were transformed with various lambda subclones (pL series) and *E. coli *subclones (pE series) described previously [22,32]. Plasmid pUC19 that does not contain the recognition sequence was used as control. To obtain non-methylated plasmids, all the plasmids were harvested from strain DB24 [33]. Non-methylated plasmids were isolated from strain DB24 [33].

## Authors' contributions

MW carried out the sequence alignment, immunoassays, complementation tests and drafted the manuscript. JR carried out the plasmids transformation method, interpreted the RM search computer program data and helped to draft the manuscript. Both authors read and approved the manuscript.

## Supplementary Material

Additional file 1 **Complementation analysis between the EcoAI and EcoAO83I subunits**. The complementation test was performed in strain BL21(DE3) harbouring plasmids with EcoAI and EcoAO83I genes ^a, b^. λ.0, λ.A and λ.C4/1 were produced as described in the legend to Table 3. λ of double specificities λ.C4/1 pJP21 and λ.C4/1 pJP24 were produced on clones ^a ^and ^b^, respectively. Click here for file

Additional file 2 **Plasmid restriction test for the EcoAO83I R-M system**. EOT values of each plasmid and presence (+) or absence (-) of the recognition site, GGA(8N)ATGC. Number of recognition sites is in parenthesis. Click here for file
