# Supplementary material for: Characterization of a restriction modification system from the commensal Escherichia coli strain A0 34/86 (O83:K24:H31)
Source: BMC Microbiol. 2008 Jun 27;8:106. doi: 10.1186/1471-2180-8-106 (PMC2481252; doi:10.1186/1471-2180-8-106)
Supplement: Additional file 1 — Complementation analysis between the EcoAI and EcoAO83I subunits. The complementation test was performed in strain BL21(DE3) harbouring plasmids with EcoAI and EcoAO83I genes a, b. λ.0, λ.A and λ.C4/1 were produced as described in the legend to Table 3. λ of double specificities λ.C4/1 pJP21 and λ.C4/1 pJP24 were produced on clones a and b, respectively. [file 1471-2180-8-106-S1.doc]

**Additional file 1** Complementation analysis between the EcoAI and EcoAO83I subunits

| **Plasmid (*hsd* genes)** | **relevant r-m**  **phenotype** | **λ.0** | **λ.A** | **λ.C4/1** | **λ.C4/1**  **pJP21** | **λ.C4/1**  **pJP24** | **λ.pJP1**  **= λ.A** |
| --- | --- | --- | --- | --- | --- | --- | --- |
| **none** | r^-^m^-^ | 1 | 1 | 1 | 1 | 1 | 1 |
| **C4/1 (*hsdR,M,S* EcoAO83I)** | r^+^m^+^ **_EcoAO83I_** | 8x10^-5^ | 10^-4^ | 1 | 0.9 | 1 | 10^-4^ |
| **^a^ C4/1 + pJP21 (*hsdR,M,S* EcoAO83I**  **+ *hsd M,S* EcoAI)** | r^+^m^+^ **_EcoAO83I_**  r^+^m^+^ **_EcoAI_** | 5x10^-5^ | 10^-4^ | 2x10^-2^ | 0.9 | 1.0 | 2x10^-4^ |
| **^b^ C4/1 + pJP24 (*hsdR,M,S* EcoAO83I**  **+ *hsdS* EcoAI)** | r^+^m^+^ **_EcoAO83I_**  r^+^m^+^ **_EcoAI_** | 2x10^-2^ | 5x10^-2^ | 2x10^-1^ | 1.0 | 1.0 | 10^-2^ |
| **pFFP30 (*hsdR, M,S* EcoAI)** | r^+^m^+^ **_EcoAI_** | 3x10^-2^ | 1.0 | 4x10^-2^ | 0.9 | 0.6 | 1.0 |

The complementation test was performed in strain BL21(DE3) harbouring plasmids with EcoAI and EcoAO83I

genes ^a, b^. λ.0, λ.A and λ.C4/1 were produced as described in the legend to Table 4. λ of double specificities λ.C4/1 pJP21 and λ.C4/1 pJP24 were produced on clones ^a^ and ^b^, respectively
