# Supplementary material for: Characterization of a restriction modification system from the commensal Escherichia coli strain A0 34/86 (O83:K24:H31)
Source: BMC Microbiol. 2008 Jun 27;8:106. doi: 10.1186/1471-2180-8-106 (PMC2481252; doi:10.1186/1471-2180-8-106)
Supplement: Additional file 2 — Plasmid restriction test for the EcoAO83I R-M system. EOT values of each plasmid and presence (+) or absence (-) of the recognition site, GGA(8N)ATGC. Number of recognition sites is in parenthesis. [file 1471-2180-8-106-S2.doc]

## Additional file 2 - Plasmid restriction test for the EcoAO83I R-M system

__________________________________________

Plasmids EOT Recognition

Sequence (# of sites)

__________________________________________

pUC19 1.0 -

pL1 1x10^-3^ + (4)

pL2 8x10^-3^ + (2)

pL3 2x10^-2^ + (1)

pL4 1x10^-3^  + (3)

pL5 2x10^-3^ + (3)

pL6 2x10^-3^ + (2)

pL8 2x10^-2^ + (1)

pL9 0.1 + (1)

pE2 0.7 -

pE3 4x10^-2^  + (1)

pE4 0.7 -

pE5 0.1 + (1)

pE6 0.1 + (1)

pE8 1.0 -

pE9 0.7 -

pE10 0.7 -

pE11 0.7 -

pE12 0.7 -

pE14 2x10^-2^  + (2)

pE15 0.7 -

pE16 1.3 -

pE17 3x10^-3^  + (1)

pE18 0.7 -

pE19 0.7 -

pE22 8x10^-2^ + (1)

pE23 4x10^-2^ + (1)

pE24 1.4 -

pE25 5x10^-2^  + (1)

pE26 0.1 + (1)

pE28 1.3 -

pE29 4x10^-2^  + (1)

pE31 0.8 -

pE32 3x10^-3^  + (3)

pE33 1.6 -

pE38 1.7 -

pE44 0.7 -

pEco377I 4x10^-3^ + (1)

_________________________________

EOT values of each plasmid and presence (+) or absence (-) of the recognition site, GGA(8N)ATGC. Number of recognition sites is in parenthesis.
